# Epilepsy and exercise: a narrative review on the crucial role of neurosteroids in modulating GABAergic neurotransmission

**DOI:** 10.1093/braincomms/fcag237

**Published:** 2026-06-22

**Authors:** Roberto Bonanni, Umberto Sansone, Ida Cariati, Alessandro Gaeta, Lilian Juliana Lissner, Veronica Alfano, Eleonora Palma, Gabriele Ruffolo, Pierangelo Cifelli

**Affiliations:** Department of Human Science and Promotion of Quality of Life, San Raffaele Open University, 00166 Rome, Italy; Department of Clinical Medicine, Public Health, Life and Environmental Sciences, University of L’Aquila, 67100 L’Aquila, Italy; Department of Systems Medicine, ‘Tor Vergata’ University of Rome, 00133 Rome, Italy; Department of Physiology and Pharmacology, Laboratory Affiliated to Pasteur Institute Italy, Cenci Bolognetti Foundation, University of Rome Sapienza, 00185 Rome, Italy; Department of Physiology and Pharmacology, Laboratory Affiliated to Pasteur Institute Italy, Cenci Bolognetti Foundation, University of Rome Sapienza, 00185 Rome, Italy; Department of Human Science and Promotion of Quality of Life, San Raffaele Open University, 00166 Rome, Italy; InterInstitutional Multidisciplinary Biobank (BioBIM), IRCCS San Raffaele Roma, 00166 Rome, Italy; Department of Physiology and Pharmacology, Laboratory Affiliated to Pasteur Institute Italy, Cenci Bolognetti Foundation, University of Rome Sapienza, 00185 Rome, Italy; Department of Physiology and Pharmacology, Laboratory Affiliated to Pasteur Institute Italy, Cenci Bolognetti Foundation, University of Rome Sapienza, 00185 Rome, Italy; IRCCS San Raffaele Roma, 00166 Rome, Italy; Department of Human Science and Promotion of Quality of Life, San Raffaele Open University, 00166 Rome, Italy

**Keywords:** epilepsy, exercise, GABA, neurosteroids, neurophysiology

## Abstract

Epilepsy is a disorder of the central nervous system characterized by the onset of seizures that significantly worsens the independence and quality of life of people affected. Although significant progress has been obtained in the research and development of pharmacological approaches able to reduce the frequency of seizures, a significant number of patients with epilepsy remain drug-resistant, highlighting the need for further research aimed at understanding the physiological mechanisms underlying neuronal excitability. In this regard, neurosteroids have been identified as key molecules in neuronal excitability due to their ability to influence GABAergic transmission paving the way for studies and research to better understand their role in epileptic mechanisms. Interestingly, some evidence has shown that moderate to intense physical exercise can influence neurosteroid synthesis, such as allopregnanolone, pregnenolone and tetrahydrodeoxycorticosterone, suggesting a therapeutic potential for physical activity in the context of epilepsy. In fact, the most recent clinical evidence has prompted a re-evaluation of the role of exercise in epilepsy, shifting from a risk factor to a non-pharmacological tool able to improve cognition and quality of life in people with epilepsy. This narrative review of the literature focuses on the role of exercise as a positive modulator of neurosteroid synthesis, as crucial GABAergic modulators, and its use as a promising non-pharmacological strategy for epilepsy.

## Teaser

Bonanni *et al*. report that physical exercise may influence seizure susceptibility by modulating neurosteroid synthesis and, consequently, GABA transmission in the CNS. Indeed, experimental and clinical studies reveal that exercise enhances neurosteroid production, thereby improving inhibitory balance and suggesting a potential complementary strategy for the management of epileptic patients.

## Graphical Abstract

**Graphical Abstract:**
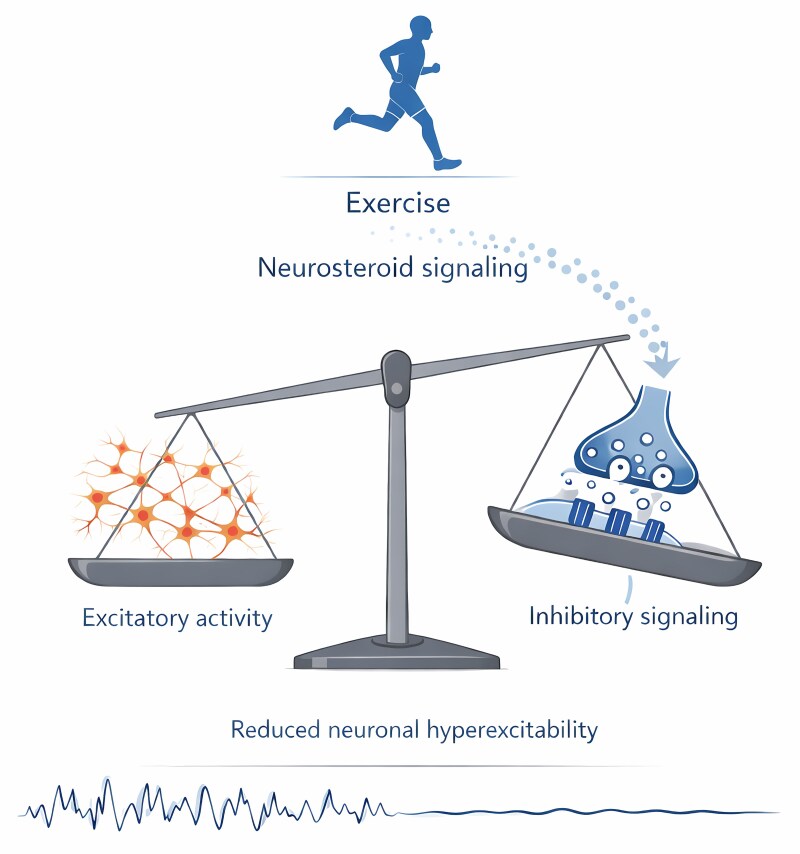

## Introduction

Epilepsy is one of the most widespread neurological diseases globally, with an estimated prevalence of ∼50 million people affected worldwide.^1^ It is defined as a chronic neurological disorder characterized by a persistent predisposition to generate epileptic seizures. These seizures are transient events caused by abnormal electrical activity in the brain, where excessive and synchronized neuronal discharges occur within the cerebral cortex.^2^ Symptomatically, during these events, we can have different manifestations that range from mild sensory alterations to involuntary violent movements including dangerous loss of consciousness. Therefore, this disorder involves important neurobiological, cognitive, psychological and social consequences that can radically impact the quality of life of affected individuals.^3^

Despite over 30 antiepileptic drugs (ASMs) currently in use, seizure control remains elusive and difficult to manage for approximately one-third of patients with epilepsy.^4^ This form of drug resistance, often related to focal epilepsies, significantly debilitates affected patients, impacting their living conditions.^5^ Sometimes drug resistance may result from maladaptive alterations in neuronal circuitry and receptor expression that compromise drug responsiveness.^6^ It has also been shown that the onset of alterations of γ-aminobutyric acid (GABA) receptor type A (GABA_A_R) function can support epileptic activity, influencing the regulation of inhibitory activity even in treated patients.^7,8^ However, even when treatments are effective, ASMs frequently cause important side effects, including impaired cognitive function, mood instability, and systemic side effects that significantly impact health status and quality of life.^9^ Therefore, the development and implementation of complementary strategies and interventions that go beyond the most widespread traditional pharmacological paradigms, such as cross-sectional lifestyle interventions, appear to be a priority line of research.^10^

Neurosteroids are a class of endogenous steroids synthesized by the central nervous system (CNS) that have the ability to exert rapid non-genomic effects on neuronal excitability.^11^ In relation to their diverse properties and specific molecular targets, they can have a modulatory effect on seizures of both inhibitory and excitatory nature.^12^ Allopregnanolone is currently the most studied anticonvulsant neurosteroid, which carries out its protective activity by functioning allosterically as a potent positive modulator of GABA_A_R_s_.^13^ Differently, compounds such as pregnenolone sulphate could inhibit GABAergic receptors and enhance the activity of N-methyl-d-aspartate (NMDA) receptors, thus playing a proconvulsant role.^14^ Moreover, in addition to their well-characterized actions on GABA_A_R_s_ and NMDA receptors, several neurosteroids may also modulate AMPA receptor–mediated excitatory neurotransmission. Experimental studies have shown that sulphated neurosteroids, including pregnenolone sulphate and dehydroepiandrosterone sulphate (DHEAS), can enhance glutamatergic transmission by facilitating AMPA receptor activity. Although the precise relevance of these effects in epilepsy remains incompletely understood, modulation of AMPA receptors may contribute to the complex and sometimes bidirectional influence of neurosteroids on neuronal excitability.^14-16^ Indeed, neurosteroids can exert bidirectional and context-specific effects in epilepsy, having in fact modulatory potential on both inhibitory and excitatory pathways. They could prove to be promising tools for therapies in settings of drug resistance, or even to support traditional guidelines.^17^ This point represents an important line of research that aims to understand neurosteroidogenesis and its exogenous modulation influenced by stimuli such as physical exercise.^18^ Indeed, some authors have observed variations in the levels of specific neurosteroids in response to exercise programmes, suggesting regular physical activity as a potential non-pharmacological tool for the treatment of epilepsy.^18,19^

Although previously considered a potential risk factor for triggering epileptic seizures,^20^ exercise has now been reviewed as a safe and potentially protective factor for epileptic individuals.^19,21^ Evidence from clinical studies indicates that regular aerobic exercise may reduce seizure frequency.^22^ Multiple processes are thought to play a key role in this phenomenon, including improved cerebral perfusion, anti-inflammatory modulation and reduced oxidative stress.^23,24^ Exercise may also reduce cortical excitability and induce beneficial changes in neuroplasticity to stabilize neuronal circuits.^25^ However, there is still a lack of univocal parameters that can be used to standardize and evaluate these impacts, which should also take into account the type of exercise, duration, intensity and, above all, individual variability.^26^

Despite the growing interest in the role of neurosteroids in epilepsy and, consequently, in the potential benefits that physical activity could have on affected patients, the interaction between these fields has not yet been sufficiently explored. Particular attention should be paid to the modulatory effect of physical activity on neurosteroidogenesis and the consequent possible influence on disease progression or seizure management.

Therefore, this review aims to analyse the currently available literature on the impact of physical exercise on epilepsy, in terms of safety and efficacy, as well as on the role of neurosteroids as potential mediators of the effects of exercise on the CNS of people with epilepsy.^11,17,22,23^

## Epilepsy and neurosteroids: what is the link?

**Figure 1:**
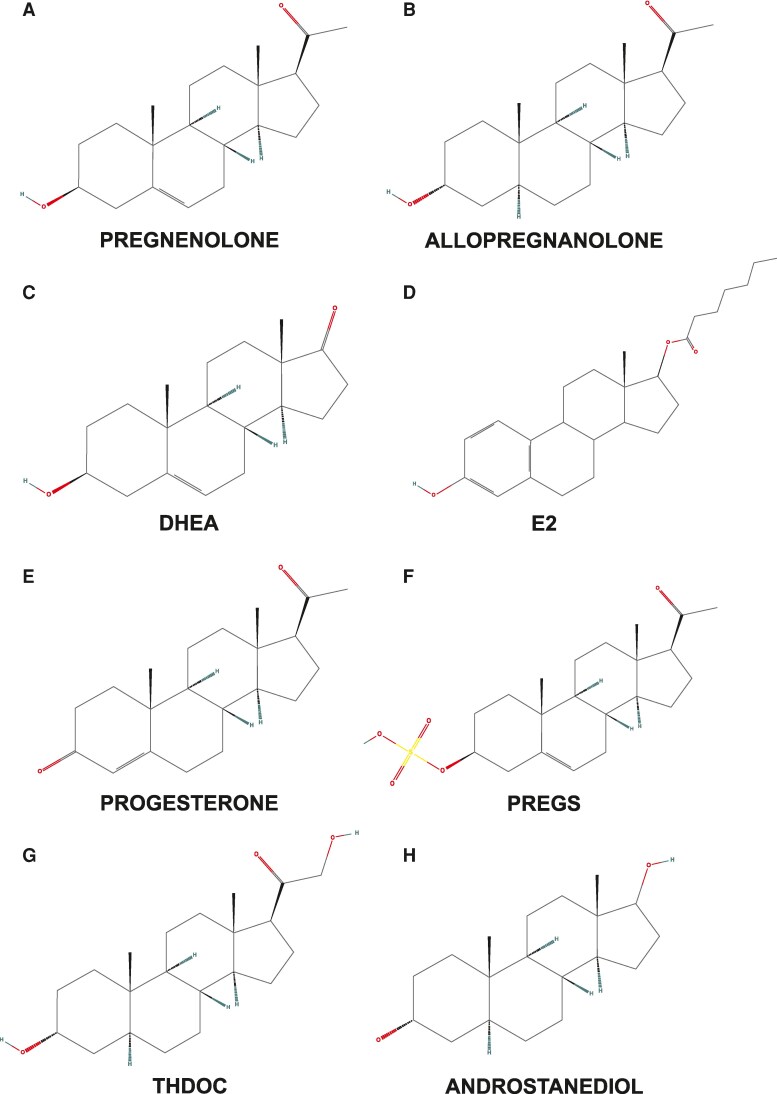
Structure of main neurosteroids involved in GABAergic modulation and epilepsy. (**A**) Pregnenolone. (**B**) Allopregnanolone. (**C**) Dehydroepiandrosterone. (**D**) Oestradiol. (**E**) Progesterone. (**F**) Pregnenolone Sulfate. (**G**) Tetrahydrodeoxycorticosterone. (**H**) Androstanediol. Images acquisition from PubChem.

Neurosteroidogenesis occurs in brain regions such as the cortex, hippocampus and amygdala, where they play a crucial role in synaptic plasticity and cognitive function.^11^ Due to their ability to influence neuronal excitability, neurosteroids have long been considered key contributors to seizure susceptibility and epileptogenesis. At the same time, they also participate in fundamental physiological processes such as neurogenesis and myelination,^27^ highlighting their crucial role in neuronal excitability.^28^ Fig. 1 shown the structure of the main neurosteroids involved in GABAergic modulation and epilepsy.

### Pregnenolone

Pregnenolone is the major precursor of neurosteroids in their biosynthetic pathway. It is synthesized in the mitochondria using cholesterol as a substrate and plays the role of progenitor for numerous neurosteroids that are formed from it, including progesterone, allopregnanolone and dehydroepiandrosterone (DHEA).^29-31^ The biological relevance of pregnenolone in this context lies mostly in its crucial role in neurosteroidogenesis and therefore in its influence on neuromodulatory balance, since in terms of direct activity on the main neurotransmitter receptors it seems to play a minor role.^29-31^

In fact, classical receptor systems such as GABA_A_ or NMDA receptors are not significantly modulated by pregnenolone at molecular level. However, this precursor, acting through mechanisms that are not yet fully understood, can influence intercellular signalling and synaptic plasticity.^29-31^

There are also suggestions from some studies that pregnenolone, by converting to active metabolites such as pregnenolone sulphate and allopregnanolone, can potentially influence neuronal resilience and cognitive processes, albeit indirectly.^30,32^

The role played by pregnenolone in epilepsy is currently poorly understood and still under study. Preclinical models suggest that the context defines its effects on seizure susceptibility. Administration of pregnenolone in some mouse models has shown proconvulsant activity, thought to be due to its conversion to excitatory derivatives such as pregnenolone sulphate.^30^ Some studies instead describe anticonvulsant effects probably due to downstream conversion to allopregnanolone which could increase GABAergic activity.^29,31^

To date, the impact of pregnenolone in patients with epilepsy is explored by minimal clinical data, as preliminary studies in humans have mostly focused on its effects on mood and cognition. Therefore, both the relationship between endogenous pregnenolone levels and epileptogenic mechanisms and its potential role in seizure control remain poorly explored.^29,30,32^

In conclusion, the influence of pregnenolone on epileptic activity is exerted by its role as a precursor of neurosteroids with the most significant effect in this regard. Furthermore, its effects appear to be strongly dose-dependent, metabolite-specific and model-sensitive, thus requiring further investigation to place this ambivalent precursor in the broader puzzle of neurosteroid-based seizure modulation.^29-32^

### Allopregnanolone

One of the most studied neurosteroids in the context of epilepsy is allopregnanolone (3α-hydroxy-5α-pregnan-20-one). This neuroactive steroid is synthesized in the brain by the enzymes 5α-reductase and 3α-hydroxysteroid dehydrogenase from progesterone, of which it is therefore a metabolite.^33^ This neurosteroid acts as a potent modulator of inhibitory neurotransmission, thus showing a high neuroactive efficacy, more marked than that of progesterone.^33^

Its neuromodulatory action is realized through its ability to allosterically potentiate GABA_A_Rs by binding to distinct sites of the receptor complex, thus potentiating the effect of endogenous GABA by increasing the influx of chloride into neurons.^33,34^ This leads to a stabilization of neural circuits, thanks to the reduction of excitability due to hyperpolarization. Specifically, extrasynaptic GABA_A_R_s_ are preferentially involved in this phenomenon, which play a key role in the regulation of global neuronal excitability as they are responsible for tonic inhibition.^33,35^

Consistent with what has been said so far, it is not surprising that allopregnanolone has shown marked anticonvulsant properties in preclinical models of epilepsy. In fact, it seems that its administration can reduce the frequency and severity of seizures in several types of epileptic animal models, including pentylenetetrazole (PTZ)-induced seizures, kindling models and genetic models of epilepsy.^35-37^ The anticonvulsant effects observed seem to be dose-dependent and sometimes comparable in efficacy to those of barbiturates and benzodiazepines, even though with different side effect profiles.^35-38^

Ganaxolone, designed as a synthetic analogue of allopregnanolone, mimics its action while improving its pharmacokinetics. This exogenous synthetic neuroactive steroid has been clinically tested in various types of drug-resistant epilepsies and early-stage studies suggest that its administration could prove to be an effective adjunctive treatment in refractory focal seizures and paediatric epileptic syndromes, although further large-scale studies to confirm its efficacy and safety are currently lacking.^34-36^

Thus, the pharmacodynamic profile of allopregnanolone is of interest for studies aimed at developing new therapies to treat seizure disorders with altered inhibitory tone or drug-resistant epilepsies through neurosteroid pathways.^34,37^ In this context, allopregnanolone represents a paradigmatic example of an endogenous neurosteroid capable of modulating GABAergic activity and therefore directly influencing epileptic activity.^33,34,36,37^

### Dehydroepiandrosterone

Dehydroepiandrosterone (DHEA) is a neurosteroid that is mainly synthesized in the adrenal cortex and in the brain. In the CNS, it plays both the role of precursor for some androgens, oestrogens and other steroids and exerts its own independent neuromodulatory activity.^39^ DHEA and its sulphate ester (DHEAS) act in the CNS where they play a regulatory role that has effects on neuronal excitability, cognitive processes and mood regulation.^39,40^

In the context of the CNS, DHEA appears to act as a weak negative allosteric modulator of GABA_A_R_s_, thus potentially reducing inhibitory tone if present at high concentrations. Its ability to potentiate NMDA receptors has also been described, potentially enhancing glutamatergic transmission and therefore the excitatory impulse.^39,41^ However, these effects appear to be dose-dependent and context-sensitive, as neuroprotective and anticonvulsant properties of this neurosteroid have been described in experimental studies.^39,41,42^

DHEA has demonstrated significant anticonvulsant effects in preclinical models of epilepsy, including iron-induced seizures that mimic post-traumatic epilepsy and chemically induced seizures such as those triggered by pentylenetetrazol (PTZ). In fact, DHEA administration in these studies was associated with a reduction in seizure frequency and severity.^39,42,43^ These effects can be partially attributed to the antioxidant properties of DHEA and its modulation of some neurotrophic signalling pathways.^39,42,43^ However, there are some examples that report proconvulsant effects associated with the administration of DHEA and its metabolites at high doses.^39,44^

Due to this modulatory duality, DHEA and its potential effects in epileptic patients are not yet fully understood, with limited and inconclusive experimental results. Overall, the effects of DHEA are not yet fully understood, highlighting the need to further investigate its neuromodulatory profile and study its possible applications in the context of epilepsy.^39,40,42,44^

### Oestradiol (E2)

E2, the most potent natural oestrogen, appears to be involved in the regulation of reproductive, cognitive and neurophysiological processes. Oestradiol is synthesized both peripherally and in the CNS, where it exerts its modulatory role through multiple genomic and nongenomic mechanisms, influencing neuronal excitability, synaptic function and neuroplasticity.^45-47^

At the molecular level, oestradiol exerts pleiotropic effects both by binding to nuclear oestrogen receptors (ER) α and β, thus influencing gene transcription, and by acting as a ligand for membrane receptors that, once activated, trigger rapid intracellular signalling cascades.^45-48^ Studies demonstrate that, by upregulating the expression and function of glutamatergic NMDA receptors, E2 stimulates excitatory neurotransmission.^45-47,49,50^

Investigations into the relationship between oestradiol and epilepsy have revealed a highly context-dependent relationship. Indeed, there is clinical and preclinical evidence comparing oestradiol’s proconvulsant and anticonvulsant effects with respect to the specific brain region involved and the hormonal context.^47-49,51,52^ Consistent with what one would expect from the molecular pathways described above, studies in mouse models describe how high oestradiol levels, presumably due to the increased glutamatergic tone they induce, are associated with increased seizure susceptibility.^47,49-51^ Other studies, focused on models of chronic epilepsy, suggest a possible neuroprotective effect of oestradiol due to its promotion of synaptic remodelling and its potential to reduce neuroinflammation.^47,51^

It is known in the clinical field that the seizure pattern of women affected by epilepsy is influenced by the fluctuations of oestradiol during the menstrual cycle; in fact, the periovulatory increase of oestradiol has been associated with an increase in seizure frequency and therefore with pro-convulsive effects in specific phases.^47,48,51-53^

Overall, oestradiol appears to be a neurosteroid with a dual impact on epilepsy. On the one hand, its excitatory effects are associated with an increased risk of seizures under certain conditions. On the other hand, its potential neurotrophic and anti-inflammatory effects are of clinical interest, due to their potential therapeutic benefits when appropriately modulated. Further research is therefore needed to clarify its potential usefulness as a potential target in the management of epilepsy.^45-50,53,54^

### Progesterone

Progesterone is a steroid hormone with well-documented neuroactive properties that is synthesized both peripherally and in the CNS. This neurosteroid influences brain function both directly and indirectly and plays important roles in the regulation of neuroprotective processes and neuronal excitability.^12,33,55-58^

At the molecular level, progesterone genomically modulates the transcription of target genes by binding to intracellular progesterone receptors (PR) A and B. However, its greater neurophysiological impact is due to the characteristics of its indirect effects, mediated by its derivative allopregnanolone, known positive allosteric modulator of GABA_A_R_s_. It is through this latter pathway that progesterone indirectly enhances inhibitory neurotransmission, thus contributing to the reduction of neuronal excitability.^33,55-58^

Progesterone, in the context of epilepsy, has been widely associated with anticonvulsant effects due to its conversion to GABAergic neurosteroids. In animal models, administration of progesterone and its metabolites significantly reduced seizure susceptibility, with dose-dependent effects influenced by the subject's hormonal status.^33,55-58^

Clinically, the therapeutic potential of progesterone has been studied in the context of catamenial epilepsy, a subtype known for its exacerbations related to hormonal fluctuations in the menstrual cycle. Some clinical studies describe how, although with variable effects, progesterone supplementation, more particularly during the luteal phase, can reduce the frequency of seizures in hormone-sensitive women.^59^

Progesterone's profile thus appears to be predominantly anticonvulsant, but factors such as the rate of conversion to active neurosteroids, interactions with other hormones, and the timing of administration represent variables that influence its effects and increase the complexity of its administration and standardization for potential clinical use. Therefore, further investigation is still needed to clarify its therapeutic potential and define its optimal clinical applications.^57-59^

### Pregnenolone sulphate

Pregnenolone sulphate (PREGS) is a sulphate ester derived from pregnenolone rather than a classical metabolite, synthesized in both peripheral tissues and the CNS. This neurosteroid is distinguished from other similar compounds by its predominantly excitatory neuromodulatory properties, due to its interactions with multiple neurotransmitter systems, making it a compound of interest in the context of epilepsy.^14,60-62^

The action of PREGS is exerted through non-genomic actions. It does not bind to intracellular steroid receptors, but rather to membrane receptors, causing a subtle modulation of the cell's excitability through signal transduction. It negatively modulates GABA_A_R_s_ activity, reducing inhibitory tone, and simultaneously positively modulates NMDA receptors, promoting excitatory glutamatergic transmission. All together these mechanisms are thought to contribute to an overall increase in neuronal excitability.^14,63^

Consistent with what has been said about its effect on neuronal excitability, PREGS has been associated with proconvulsant effects in preclinical studies. Indeed, PREGS administration in animal models appears to lower the seizure threshold and facilitate seizure onset in various paradigms. However, even for this neurosteroid, the effects appear to be dose-dependent and context-specific, depending on the model's baseline excitability and the presence of other modulatory factors.^60-62,64^

It is important to emphasize, however, that PREGS does not play an entirely harmful role. In some models, low concentrations of PREGS have been linked to neuroprotective and cognitive-enhancing effects, suggesting a bidirectional, dose-sensitive profile. It should also be considered that the balance between PREGS and its parent compound, pregnenolone, and its conversion into other neuroactive metabolites also modulate its net impact on neuronal excitability.^61,64^

Currently, human data remain too sparse to permit direct clinical applications of PREGS in the treatment of epilepsy. For now, it can only be assumed that, given its excitatory profile, therapeutic manipulation of PREGS would likely focus on strategies aimed at limiting its levels or activity, especially in patients sensitive to its concentrations in their seizure susceptibility. Given the scarcity of recent investigations, further research is needed to clarify its role in human epileptogenesis and its interaction with other neurosteroids.^60,62,64^

### Tetrahydrodeoxycorticosterone

This neurosteroid exerts its effect by binding to the allosteric sites of GABA_A_R_s_, increasing GABA-mediated chloride influx, thus promoting neuronal inhibition. Its modulatory effect is particularly pronounced under conditions of increased neurosteroidogenesis, such as acute stress or hormonal fluctuations.^65^ The action of tetrahydrodeoxycorticosterone (THDOC) is rapid, as it does not require transcriptional changes, qualifying it as a fast-acting neuromodulator capable of positively regulating GABA-evoked currents.^65,66^

THDOC has been described in preclinical models of epilepsy as having strong anticonvulsant effects. Indeed, the literature describes how exogenous THDOC administration increases the seizure threshold and reduces seizure frequency in various rodent models, including those induced by PTZ and kindling protocols.^67^

Endogenous regulation of THDOC levels may also play a role in the pathophysiology of epilepsy itself. It has been hypothesized that deficits in neurosteroid synthesis or the expression of specific GABA_A_R_s_ subunits may reduce the brain's sensitivity to THDOC, thus contributing to increased seizure susceptibility. Furthermore, fluctuations in THDOC synthesis during stress or hormonal changes could further influence neuronal excitability.^68^

Despite solid and promising preclinical evidence, clinical data on THDOC in epilepsy remain scarce. Noteworthy, a reduced serum concentration of THDOC was detected during the menstrual cycle of women with catamenial epilepsy, highlighting the importance of neuroendocrine variations in epileptic syndromes.^69^ However, further research is hampered by its rapid metabolism and low basal concentrations, which increase the difficulty of measuring its plasma or cerebrospinal fluid levels in humans. In summary, THDOC is a potent endogenous modulator of the GABA_A_R_s_ with well-established anticonvulsant activity in animal models. Its relevance in human epilepsy, however, is supported by a plausibility that is currently only mechanistic, and further translational research is therefore necessary to fully clarify its action mechanism.^67,70^

### Androstanediol

Androstanediol is a neuroactive metabolite derived from dihydrotestosterone (DHT) through enzymatic reduction by 3α-hydroxysteroid dehydrogenase in the CNS. This conversion produces 3α-androstanediol, a compound capable of rapidly influencing neuronal activity through non-genomic mechanisms. Like other 3α-reduced neurosteroids such as allopregnanolone and THDOC, androstanediol is recognized as a positive modulator of GABA_A_R_s_ and thus contributes to the regulation of neuronal excitability.^71,72^

At the molecular level, androstanediol exerts its effects by binding to allosteric sites on GABA_A_R_s_, thereby increasing GABA-mediated chloride influx and thus promoting neuronal inhibition. Its action is stereospecific and varies across brain regions, appearing to have a greater impact on limbic structures such as the hippocampus and amygdala, key areas involved in emotional regulation and the generation of epileptic seizures. Compared to other GABAergic neurosteroids, androstanediol exhibits similar pharmacodynamics but slightly lower potency, suggesting a modulatory rather than dominant role in inhibitory signalling.^73^

Currently, preclinical research, although limited, seems to suggest that androstanediol may exert promising anticonvulsant activity in animal models.^74^ Experimental studies report a reduction in seizure frequency and an increase in seizure threshold in paradigms such as PTZ-induced seizures and electrical stimulation. These effects appear, as already highlighted for several neurosteroids previously discussed here, to be dose-dependent and context-sensitive.^75^

Clinical data on androstanediol are currently insufficient to test its therapeutic use in epileptic patients. Possible connections between its physiological endogenous fluctuations and its potential contribution to sex hormone-related seizure susceptibility have been hypothesized, particularly in hormone-sensitive forms such as catamenial epilepsy, but at present, there are insufficient data to consolidate this link.^76^

In conclusion, consistent with our current knowledge of the link between androstanediol and epilepsy, we can only state that, although preclinical evidence suggests promising anticonvulsant potential, the compound's pathophysiological role in humans remains speculative, as does its potential future therapeutic use. Further investigations are needed to clarify its relevance within the broader neurosteroid–epilepsy interface and to define its possible role in epilepsy therapy.^71,73,75^

### Exercise modulates neurosteroid levels

Several evidences have shown the efficacy of exercise in influencing GABA concentration, highlighting increases in brain levels of the neurotransmitter in response to exercise programmes. Therefore, exercise could significantly influence GABA-mediated neuronal excitability, but the effects of regular exercise on GABAergic circuits remain still partially unknow.^77-79^ In this regard, exercise has been recognized as a powerful modulator of neurosteroidogenesis, capable of influencing mood, cognitive functions, and pain, but the evidence currently available on the influence of exercise on neurosteroid signalling is still limited.^27^ However, some important evidence confirms the modulatory role of exercise on neurosteroid levels, suggesting their role as potential mediators of the analgesic and anticonvulsant effects of exercise.

Significant evidence of the effects of exercise on progesterone and allopregnanolone levels was provided by Aoyama *et al*., which investigated neurosteroid levels in adult and elderly male Winstar rats trained on treadmills. In detail, the animals were divided into a control group, that did not undergo exercise, a low-intensity exercise group, and a high-intensity exercise group. Neurosteroid and 5α-reductase levels were quantified in the brain, spinal cord, and plasma 20 min after the experimental procedure. Spinal and plasma levels of progesterone and allopregnanolone did not show significant exercise-induced changes, while some important fluctuations were observed in brain levels.

Interestingly, brain progesterone levels were not influenced by physical activity, but allopregnanolone levels were significantly higher in adult rats of the high-exercise group and in aged rats of the low-exercise group, suggesting the need for age-appropriate interventions to optimize neurosteroid levels. In agreement, 5α-reductase levels were significantly higher in elderly low-exercise rats and showed a tendency to increase in adult high-exercise rats, which, however, did not reach statistical significance.^80^

Interestingly, exercise is known to represent a stress factor able to stimulate, in a dose- and intensity-dependent manner, the production of neurosteroids, confirming the observations of Aoyama and colleagues on the effects of high-intensity exercise.^81^ In fact, other preclinical evidence has investigated the levels of neurosteroids, such as the enzymes involved in their biosynthesis, in rodent models undergoing forced swimming stress. This evidence found a statistically significant increase in both transcripts and protein products of the two isoforms of 5α-reductase, 5α-R1 and 5α-R2, in the prefrontal cortex of mice undergoing acute swimming stress compared to control mice, suggesting an increase in allopregnanolone production following exercise stress.^81,82^

Further preclinical evidence of the ability of physical exercise to modulate neurosteroid production was provided by Chai and colleagues, who suggested pregnenolone as a potential mediator of the protective effects of exercise against fluoride-induced neurotoxicity. In fact, animals treated with sodium fluoride (NaF) showed deterioration of the mitochondrial membrane and myelin sheath, a reduced number of hippocampal neurons, and a decline in memory and learning. However, exposure to exercise on a treadmill preserved mitochondrial structure and myelin sheath integrity, highlighting the ability of exercise to counteract NaF-induced neurotoxicity. Noteworthy, the NaF treatment was associated with lower hippocampal expression of pregnenolone, while, the content of this neurosteroid was significantly higher in trained mice, suggesting a role as a biomarker reflecting the alleviating effects of exercise on hippocampal damage.^83^

The efficacy of exercise in enhancing the synthesis of anticonvulsant neurosteroids has been suggested by Reddy *et al*., which studied the role of deoxycorticosterone (DOC) and its metabolite THDOC in the regulation of susceptibility to stress-induced seizures. The authors used male Sprague Dawley rats subjected to forced swimming and subsequently treated with PTZ to induce seizures. Interestingly, the threshold of susceptibility to PTZ-induced seizures was significantly increased in rats undergoing acute swimming stress, concomitant with an increase in plasma THDOC levels. Importantly, pre-treatment of animals with finasteride, a 5α-reductase inhibitor, 90 min before swimming stress did not significantly alter plasma THDOC levels in non-stressed rats, whereas in mice undergoing forced swimming, it reduced the seizure threshold and prevented the increase in plasma THDOC. Since finasteride is a 5α-reductase inhibitor capable of blocking the conversion of DOC to 5α-dihydrodesoxycorticosterone, the metabolite that is reduced to form THDOC, these results suggested that the anticonvulsant effects of acute swimming stress may be mediated by increased DOC synthesis or availability. In fact, systemic administration of DOC increased both the threshold of PTZ-induced seizures and plasma THDOC levels, suggesting a modulatory role on GABA_A_R_s_ through its conversion to THDOC.^84^

Therefore, preclinical evidences confirm a modulatory role of physical exercise on neurosteroid synthesis, selecting this class of cholesterol derivatives as potential effectors of the benefits of exercise on the CNS. The evidences mentioned above suggest an effect dependent on both age and exercise parameters, such as duration and intensity, but further research is needed to clarify the physiological mechanisms underlying physical activity-induced neurosteroid synthesis.

## Exercise and epilepsy: what does the clinical evidence say?

Among the molecular mechanisms underlying the beneficial effects of exercise on the CNS, increased production of neurotrophic factors, stimulation of hippocampal neurogenesis, increased neuronal vascularization, and others have been proposed.^85-87^ Notably, there is evidence supporting GABAergic plasticity resulting from chronic exercise, suggesting a role in the management of epilepsy.^77,88^

In recent years, the role of exercise in individuals with epilepsy has been significantly reconsidered, shifting from a potential risk factor for triggering seizures to a recommended non-pharmacological therapeutic strategy.^89^ In fact, studies aimed at clarifying the effects of physical exercise programmes in individuals with epilepsy have not found significant increases in the frequency of seizures during the interventions.^25^ Furthermore, relevant evidence has shown that combined physical exercise can improve executive function, attention and language skills in adults with epilepsy, representing a valuable tool for promoting cognitive improvement in individuals with this condition.

In this regard, in 2020, Feter *et al*. published the results of a randomized clinical trial (RCT) in *Epilepsia* journal, which investigated the effect of a 12-week individualized and supervised exercise programme on cognitive function in people with epilepsy. Specifically, 21 subjects with epilepsy were randomized into a control group (CG, *n* = 10), which maintained normal daily activities, and an exercise group (EG, *n* = 11), which received a combined exercise programme consisting of warm-up, aerobic training, strength training, and stretching, structured into two 60-min sessions per week. At the end of the experimental period, the authors found a significant improvement in verbal fluency and cognitive function in the group of participants who underwent physical exercise, providing the first evidence of its efficacy in improving executive function in people with epilepsy.^90^

One year later, the same authors published in *Epilepsy Behavior* the results of an RCT conducted on a Brazilian population with epilepsy undergoing a structured 12-week exercise programme, aimed to evaluate the effects on health of people with epilepsy. Participants were randomized into a control group (CG) and an exercise group (EG), which underwent a structured and supervised programme consisting of warm-up, aerobic training, strength training and stretching exercises for ∼1 h on 2 days per week, for a total of 24 training sessions. Although one EG subject experienced eight seizures, there were no statistically significant differences in seizure frequency between the exercise group and the control group. In addition, quality of life scores showed significant improvement in the domains of seizure preoccupation, cognitive function, and overall quality of life, suggesting that exercise is a welcome initiative for people with epilepsy.^91^

Similar observations were reported by Kumar and colleagues, which conducted an RCT on 117 individuals with generalized or focal epilepsy, randomly divided into a control group (CG, *n* = 59) and an exercise group (EG, *n* = 58). Specifically, the intervention group was recommended to perform at least 150 min of moderate/intense aerobic physical activity per week, for 12 weeks, monitored through a smartphone app. At the end of the study, a non-significant trend towards improvement in quality of life and seizure frequency was recorded, suggesting the need for further studies with longer observation periods. Therefore, although no significant effects induced by exercise were observed, the observations of Kumar *et al*. did not lead to the conclusion that physical exercise should be contraindicated in people with epilepsy, as it does not affect either the frequency of seizures or the severity of the condition.^92^

A valuable contribution to clinical research in the field of epilepsy was provided by Ibañez-Micó *et al*., which conducted the first prospective, randomized, controlled study on the effect of a physical education programme in children with drug-resistant epilepsy.^93^ In detail, 29 children were randomized into an exercise group (EG, *n* = 14), which adhered to a programme of at least 1 h of aerobic activity 3 days per week, in addition to that performed at school, monitored by a wearable device for 6 months, and a control group (CG, *n* = 15). Interestingly, adherence to physical activity in the control group was not significantly different between baseline and end-of-study values; while in the intervention group, physical activity increased from 1 h per week to 5.82 h per week, highlighting the great interest of children with epilepsy in physical activity. Notably, no significant differences in seizure frequency were found between the two groups, but a negative correlation was found between hours of physical activity and seizure frequency, demonstrating the effectiveness of physical activity in the management of individuals with drug-resistant epilepsy.^93^

Therefore, the evidence currently available supports the use of physical exercise in the epileptic population, because there is no increase in the number of seizures in individuals who adhere to regular physical activity programmes. Overall, clinical studies have found benefits from physical exercise in terms of improved quality of life, cognitive function and seizure frequency, although some studies have not found significant differences for these endpoints (Table 1). Further studies with larger populations and longer follow ups are needed to determine the real benefits of physical activity in individuals with epilepsy. In addition, it is important to clarify the differences between voluntary physical activity and structured exercise, assessing the influence of parameters such as type, intensity, and duration on seizure frequency and quality of life improvement.

**Table 1:** Clinical evidence on the effects of exercise in people with epilepsy

Population	Intervention	Comparator	Outcomes	Reference
EG: 11 subjects (male *n* = 4, female *n* = 7), average age 37.1 years	Supervised exercise programme structured into two session per week of 60 min each, featuring warm-up, aerobic training, strength training, and stretching	CG: 10 subjects (male *n* = 4, female *n* = 6), average age 39.7 years, who maintained their normal daily activities	Improvement in verbal fluency and cognitive function	^90^
EG: 11 subjects (male *n* = 4, female *n* = 7), average age 37.1 years	Supervised exercise programme structured into 1-h sessions, twice a week, for a total of 24 training sessions, featuring warm-up, aerobic training, strength training and stretching	CG: 10 subjects (male *n* = 4, female *n* = 6), average age 39.7 years, who maintained their normal daily activities	No significative difference in seizure frequency Improvement of quality of life and cognitive function	^91^
EG: 58 subjects (male *n* = 39, female *n* = 19), average age 27.1 years	At least 150 min of moderate or vigorous aerobic physical activity per week for 12 weeks, monitored by a smartphone-based app	CG: 59 subjects (male *n* = 43, female *n* = 16), average age 26.2 years	No significant improvement in quality of life and epileptic seizure frequency	^92^
EG: 14 children with drugs-resistant epilepsy (male *n* = 5, female *n* = 9), average age 10.85 years	At least 1 h of aerobic activity, 3 days per week, in addition to that performed at school	CG: 15 children with drugs-resistant epilepsy (male *n* = 7, female *n* = 8), average age 8.73 years	Negative correlation between hours of physical activity and seizure frequency	^93^

EG, exercise group; CG, control group.

Table 1 summarizes, in PICO format, the characteristics of the main RCTs conducted on people with epilepsy undergoing physical exercise interventions.

## Conclusion

**Figure 2:**
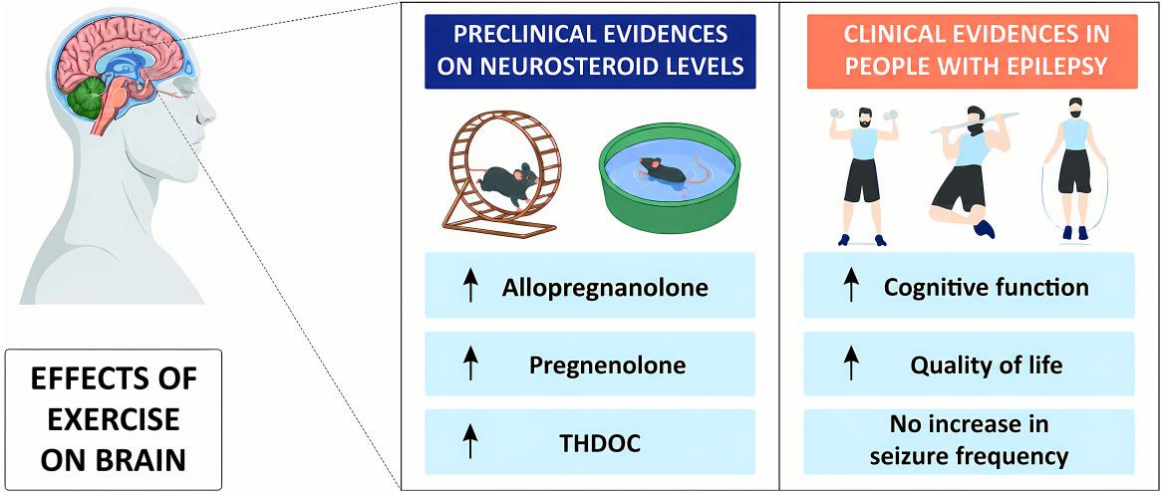
Preclinical and clinical evidence on the effects of physical exercise in the context of neurosteroid modulation and epilepsy. Schematic representation of the main findings emerging from preclinical studies, showing the modulation of key neurosteroids (allopregnanolone, pregnenolone and THDOC) following exercise or exercise-related stress paradigms, and clinical studies in people with epilepsy, highlighting improvements in cognitive function and quality of life, with no increase in seizure frequency. This figure was generated using an AI-assisted image editing workflow based on OpenAI ChatGPT image generation.

Epilepsy is a CNS disorder characterized by abnormal electrical activity, responsible for the onset of seizures that significantly reduce the independence and quality of life of affected individuals. The GABA_A_R_s_ appears to be the ideal candidate for the management of epilepsy, because the alteration of their function can promote the development of epileptic seizures. Therefore, the development of strategies capable of modulating GABAergic function could represent a valuable support for the management of people with epilepsy. In this context, neurosteroids are a class of steroids that significantly influence the metabolism and neurotransmission in the CNS. Due to their ability to regulate GABAergic activity, they have been suggested as key molecular players in epilepsy, as their synthesis in the CNS can control the frequency of seizures in epileptic animals (Fig. 2). Importantly, some preclinical evidence has shown that moderate to intense physical exercise can represent a stress factor capable of modulating neurosteroid synthesis and, consequently, GABAergic activity, suggesting its therapeutic potential for the management of individuals with epilepsy. In fact, in recent years, available clinical trials did not report any adverse effects induced by exercise in individuals with epilepsy, highlighting the safety of this non-pharmacological intervention even in patients with drug-resistant epilepsy (Fig. 2). Furthermore, some studies have found a significant improvement in quality of life and cognitive function following exercise programmes, justifying the need for further studies aimed to clarify the benefits of this non-pharmacological approach in epilepsy. Finally, some authors have reported that physical exercise may be a promising strategy to reduce the number of seizures or improving their control,^94,95^ highlighting the potential of this strategy as a non-pharmacological treatment for epilepsy. Therefore, clinical studies with large populations and longer follow-up periods are needed to clarify the benefits and safety of exercise in epilepsy, as well as the role of neurosteroids as potential mediators of the effects of exercise on GABAergic regulation.

## Data Availability

Data sharing is not applicable to this article as no new data were created or analysed in this study.
